# Recent Advances in Polysaccharide-Based Hydrogels for Tumor Immunotherapy

**DOI:** 10.3390/gels11030152

**Published:** 2025-02-20

**Authors:** Youxi Zhou, Kaizhao Chen, Hongwei Cheng, Shuaishuai Zhang

**Affiliations:** 1 Key Laboratory of Brain, Cognition and Education Sciences, Institute for Brain Research and Rehabilitation, Guangdong Key Laboratory of Mental Health and Cognitive Science, Ministry of Education, South China Normal University, Guangzhou 510631, China; 2024024674@m.scnu.edu.cn (Y.Z.); chenkaizhao787@163.com (K.C.); 2 Zhuhai UM Science & Technology Research Institute, University of Macau, Macau 999078, China

**Keywords:** polysaccharide hydrogels, immunotherapy, drug delivery, tumor microenvironment

## Abstract

Immunotherapy has revolutionized cancer treatment and led to a significant increase in patient survival rates and quality of life. However, the effectiveness of current immunotherapies is limited by various factors, including immune evasion mechanisms and serious side effects. Hydrogels are a type of medical material with an ideal biocompatibility, variable structure, flexible synthesis method, and physical properties. Hydrogels have long been recognized and used as a superior choice for various biomedical applications. The fascinating results were derived from both in vitro and in vivo models. The rapid expansion of this area suggests that the principles and uses of functionalized polysaccharides are transformative, motivating researchers to investigate novel polysaccharide-based hydrogels for wider applications. Polysaccharide hydrogels have proven to be a practicable delivery strategy for tumor immunotherapy due to their biocompatibility, biodegradability, and pronounced bioactive characteristics. This study aims to examine in detail the latest developments of polysaccharide hydrogels in tumor immunotherapy, focusing on their design, mechanism of action, and potential therapeutic applications.

## 1. Introduction

Immunotherapy has emerged as a potent therapeutic approach for cancer. The quantity of immunotherapy medications and techniques has been rising in clinical settings. Nonetheless, the regulated modulation of the immune system remains a challenge for immunotherapy. Comprehending methods to enhance the response to immunotherapy is crucial for augmenting efficacy and managing undesirable effects [1]. Advanced biomaterials such as nanoparticles is an emerging form of delivery therapy that focuses on improving the therapeutic effect while reducing side effects. A nanomaterial-based drug delivery strategy not only is an effective way to overcome chemotherapy resistance, but also plays a unique advantage in tumor immunotherapy [2,3].

Hydrogels consist of water-insoluble, crosslinked polymers. These three-dimensional (3D) polymeric gels possess hydrophilic, porous networks capable of absorbing water up to hundreds of times their dry weight [4]. Because of the adjustable structure, and the flexible synthesis method, as well as good physical and chemical properties, hydrogels have become the first choice for many applications in the field of biomedical materials [5]. Hydrogels can be classified in several ways. Hydrogels can be categorized into physical and chemical types based on their binding mechanisms. Hydrogels can be categorized as synthetic hydrogels and natural hydrogels based on the origin of their materials. The most extensively researched natural hydrogels are derived from polysaccharides and peptides [6]. Polysaccharides possess numerous exceptional physical and chemical features, including biocompatibility, biodegradability, non-toxicity, harmlessness, ease of metabolism, and the ability to be absorbed by the human body as nutrients [7]. Moreover, the sources of polysaccharides are diverse and uncomplicated, cost-effective, and conducive to large-scale manufacturing compared to alternative raw materials, hence facilitating the advancement of polysaccharide-based hydrogels [8]. Consequently, hydrogels derived from polysaccharides have been extensively utilized in numerous biomedical applications.

Numerous researchers utilize polysaccharide backbones or their derivatives, including alginic acid, cellulose derivatives, hyaluronic acid, chondroitin sulfate, chitosan, and cellulose, to fabricate hydrogels [9,10,11,12,13]. The numerous functional groups in polysaccharide structures offer accessible reaction sites for hydrogel formation, regardless of whether through physical or chemical crosslinking [14]. In recent years, a range of controlled polymer-grafted polysaccharides has been synthesized using live radical polymerization (LRP), ring-opening polymerization (ROP), and self-assembly techniques [15]. Alterations of polysaccharide hydrogels can augment their therapeutic efficacy. The incorporation of functional groups, including peptides, proteins, and antibodies, enhances the adhesion of cells and therapeutic agents, resulting in superior drug delivery and targeting. The altered polysaccharides (aminated, PEGylated, heparin; supramolecular structures comprising β-cyclodextrin) demonstrate remarkable therapeutic efficacy for cancer treatment applications [16].

Hydrogels represent a promising platform for improving the intratumoral delivery of immunotherapy. These drug delivery systems based on biomaterials are composed of a semi-solid polymer network with a high water content, which enables the localized and sustained release of various encapsulated drugs [17]. Stimuli-responsive hydrogels are particularly attractive because of their capacity to transform from liquid to semi-solid gel in response to physiological stimuli (such as pH or temperature). This characteristic allows stimuli-responsive hydrogels to be administered directly into a tumor through a syringe or catheter, eliminating the requirement for surgical implantation. Additionally, stimuli-responsive hydrogels can offer a three-dimensional scaffold-like microenvironment that attracts immune cells and promotes interactions between the immune system and entrapped tumor antigens or immunomodulators [18]. Alternatively, shear-thinning or “self-healing” hydrogels can be employed, which experience a decrease in viscosity when passing through a needle and restore their initial viscosity once the applied shear stress stops [19].

This document summarizes the recent advancements in polysaccharide-based hydrogels and their significance in tumor immunotherapy. Our review will attract researchers focused on biomaterials and cancer therapy. Despite the numerous hurdles encountered in the therapeutic application of polysaccharide hydrogels, the reported outcomes in cancer treatment, particularly in tumor immunotherapy, are remarkable. These fundamental concepts and extensive information serve as significant references for pharmacists, chemists, biological scientists, and clinical practitioners, potentially offering fresh perspectives on tumor therapy and breakthroughs in other domains.

## 2. Design and Preparation of Polysaccharide Hydrogels

Hydrogels are predominantly synthesized through crosslinking and non-crosslinking steps to establish a stable polymer network. Polysaccharide hydrogels are mainly synthesized through the crosslinking of polysaccharides, including hyaluronic acid, chitosan, and alginate, in aqueous solutions. The structural and degradation characteristics of these hydrogels can be modulated through adjustments in their formulation, material ratio, and network interconnectivity. According to the crosslinking methodology, polysaccharide-based hydrogels fall into two categories: physical and chemical networks [20].

Physical crosslinking relies on non-covalent mechanisms including ionic attractions, hydrogen bonds, polymer chain interweaving, and specific molecular recognition pairs. The reversible nature of these non-covalent associations enables responsive behavior to external stimuli [21]. This physical linking approach offers advantages in biocompatibility by avoiding chemical crosslinking agents and their potential toxic effects. In particular, hydrogel systems combining oppositely charged polysaccharides achieve stability through electrostatic forces, making them responsive to environmental pH and ionic conditions. Hydrogels with tissue specificity have been synthesized utilizing an electrostatic interaction crosslinking process, informed by the structural properties of polysaccharides [22]. Electrostatic interactions possess the following attributes: (1) Simple and gentle preparation conditions, without the incorporation of chemical crosslinkers, yield excellent biocompatibility. (2) A robust self-repair capability: During the ion crosslinking process, various divalent or multivalent metal ions can coordinate with their respective ligands, allowing for the quick and reversible binding and dissociation of these interactions. (3) Excellent conductivity: Electrostatic interactions and ionically crosslinked hydrogels substantially enhance conductivity through a charge transfer under an electric field. Electrostatic interactions often arise between cations and anions, and the primary technique for synthesizing this hydrogel is a direct electrostatic interaction between a minimum of two chemically distinct groups possessing opposite charges [23]. Natural polymers, like hyaluronic acid and alginate, carry negative charges due to carboxyl groups, while others like chitosan have positive charges due to amino groups. When these solutions with opposite charged polyelectrolytes are combined, they interact to create insoluble compounds [24]. Based on their main functional groups, natural polysaccharides employed for the construction of hydrogels can be classified into anionic, cationic, and neutral ones. Different kinds of polysaccharides with multiple structures endow them with diverse properties and the potential performance of gelatinization (Table 1).

Radical polymerization is the predominant method for chemical crosslinking, typically necessitating a chemical initiator to commence the polymerization of acrylate or vinyl polysaccharides. Chemical crosslinked hydrogels are produced not only through radical polymerization but also by establishing dynamic covalent bonds, such as Diels–Alder reactions, azide–alkyne cycloaddition (“click” chemistry), Schiff base formation, Michael addition reactions, disulfide bond formation, and borate ester crosslinking. The stability, degradability, and mechanical properties of chemical crosslinked hydrogels are better than those of physical crosslinked hydrogels. Covalent crosslinking can be divided into monomer crosslinking and polymer crosslinking. The monomer pathway from monomer to hydrogel is free radical polymerization (FRP). Common approaches for free radical polymerization encompass bulk polymerization, solution polymerization, suspension polymerization (including reverse suspension polymerization), and emulsion polymerization (including micellar polymerization) [25]. In the polymerization process, the monomer must initially be polymerized into polymer chains with the aid of a crosslinking agent prior to the occurrence of interchain crosslinking. In both natural and synthetic hydrogels, three predominant methods for polymerization exist: the first employs crosslinking agents like glutaraldehyde; the second utilizes high-energy radiation crosslinking, a direct and additive-free technique; the third incorporates chemical reactions, including the Schiff base reaction, Michael addition reaction, and aziridine cycloaddition reaction [26,27,28]. Different from the non-covalent mechanism of physical crosslinking, chemical crosslinking dependent on radical polymerization is more conducive to the production of polysaccharide hydrogels with diverse structures and complex functions.

The kinetics of the polysaccharide hydrogel formation is typically extremely rapid and the resultant polysaccharides hydrogels are robust enough to be applicable in numerous industrial and biomedical fields. However, the pharmacokinetics of the polysaccharide hydrogel release need to be adjusted and optimized according to the specific treatment situation. Sometimes, more than one pharmacokinetics model can better describe the sustained release profiles, owing to the existence of drug release mechanisms from polysaccharide hydrogels in parallel [29]. The rapid release kinetics of the drug from the polysaccharide hydrogel will lead to tissue and plasma pharmacokinetics similar to those of the free drug. This might require repeated administration to make up for the rapid clearance of the drug from the tumor, and dose adjustments based on the degree of systemic exposure and toxicity. Furthermore, the single intratumoral injection of the drug-loaded polysaccharide hydrogel for sustained drug delivery might reduce the necessity of repeated or metronomic dosing, which is common in clinical dosage algorithms [30]. Stimuli-responsive (such as pH or temperature) hydrogels and a controlled drug release have also been investigated, and this might have particular significance for the timelines inherent in the tumor therapy [22,31,32]. To evaluate the drug release from polysaccharide hydrogels, the hydrogel precursor solution with a known amount of active ingredient is placed in a mold before gelling to obtain a consistent sample morphology. The polysaccharide hydrogel loaded with the active ingredient is then dipped into a release medium such as a buffer solution and incubated under controlled conditions which simulate the intended physiological environment. Samples are taken from the release medium at predefined times and the released drug concentration is determined using suitable analytical methods such as liquid chromatography or UV spectrophotometry [33]. The rate of cumulative drug release (%) was determined as follows:

Cumulative release (%) = (Q_t_/Q_0_) × 100%

where Q_t_ is the amount of drug released from the hydrogel network at time t and Q_0_ is the initial amount of the drug-loaded hydrogel [34].

**Table 1:** The chemical structure of natural polysaccharides applied for biomedical hydrogels reported in the references.

Charge Condition	Glycosyl Skeleton	Chemical Structure	Functions and Advantages	Applications
Anionic	Hyaluronic acid	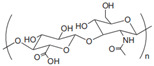	Main component of ECM; regulate cell adhesion, proliferation, and migration.	Targeted drug delivery; tissue regeneration [35,36]
	Alginate	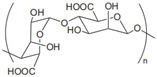	Porosity, hydrophilicity, degradability, and biocompatibility.	Tissue regeneration, drug delivery, cancer treatment, and antimicrobials [37]
	Chondroitin sulfate	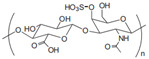	Anti-inflammatory; anti-rheumatic	Dietary supplement for joint health [6]
	Heparin	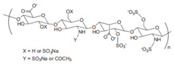	Inhibit platelet adhesion and aggregation; reduce glycocalyx damage.	Anti-coagulant; anti-atherosclerosis [38,39]
	Carrageenan	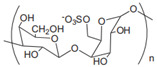	κ, ι, and λ are three kinds of carrageenan, which share different substrate specificity.	Pharmaceutical excipients: thickener; suspending agent [40]
	Fucoidan	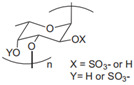	Natural ligands for selectins; anti-inflammatory, anti-coagulant, and anti-tumor.	Nutritional supplement [41,42]
	Tragacanth gum	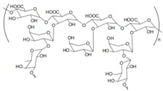	Anti-coagulant, anti-inflammatory, antiviral, anti-tumor and anti-oxidant.	Pharmaceutical excipients: thickener, emulsifier; suspending agent [43,44]
Cationic	Chitosan	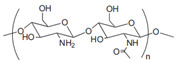	Natural cationic polysaccharides.	Drug delivery; tissue engineering; films material [45,46]
Neutral	Dextran	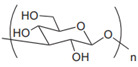	Anti-thrombotic; extend the circulation time of contrast agents by surface coating with dextran.	Substitutes for blood plasma; drug delivery; tissue engineering [47]
	Cellulose	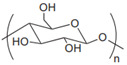	Most abundant natural polymer; the constituents of plant cell wall.	Drug delivery; tissue engineering [48]
	Pullulan	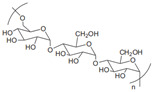	Free radical scavenger; anti-adhesion.	Drug delivery; tissue engineering [49,50]
	Cyclodextrin	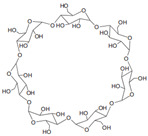	α, β, and γ are three kinds of cyclodextrin. β-cyclodextrin is widely used in drug industry.	Construction of complexes with various guest molecules [51]

## 3. Application of Polysaccharide Hydrogels

**Figure 1:**
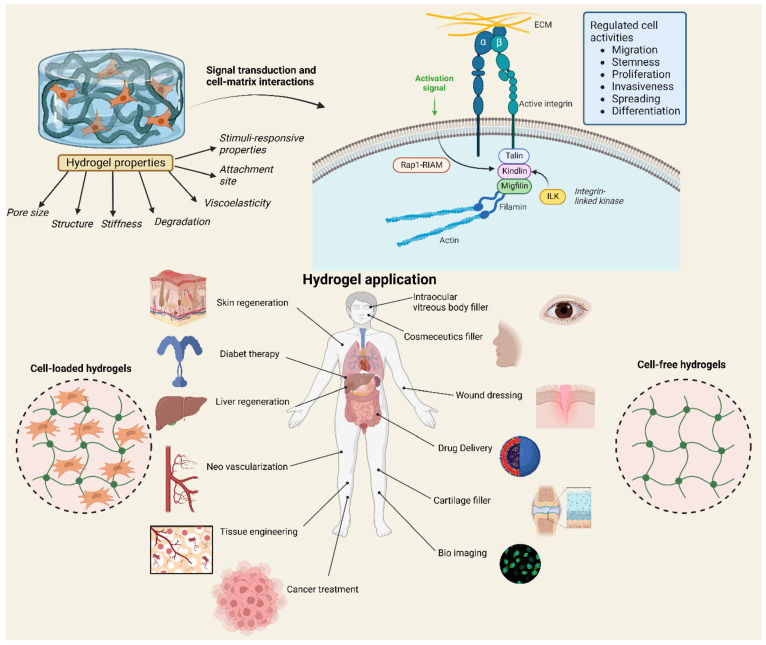
Biomedical application of polysaccharide hydrogels. The distinctive characteristics of polysaccharide-based hydrogels render them suitable for applications in skin and liver regeneration, diabetes therapy, drug delivery, cancer treatment, neovascularization, bio-imaging, tissue engineering, and wound dressings. Reproduced with authorization from Ref. [53]. Copyright 2022 Licensee MDPI, Basel. Farasati Far B and colleagues. Polymers (Basel).

Polysaccharide hydrogels have shown promising results in preclinical studies, raising hopes for their translation to clinical applications. The diverse characteristics of polysaccharide hydrogels render them suitable for possible applications in skin and liver regeneration, diabetes therapy, drug delivery, cancer treatment, neovascularization, bio-imaging, tissue engineering, and wound dressings [52,53] (Figure 1). Polysaccharide hydrogels infused with therapeutic agents and cellular constituents are presently being studied for the treatment of several ailments, including malignancies, inflammatory disorders, and infectious diseases.

Due to their distinctive physicochemical features, particularly self-healing capabilities, different polysaccharide hydrogels have been widely utilized in wound-healing and tissue-engineering applications [54,55]. Table 2 summarized a series of clinical trials of polysaccharide hydrogels (data from ClinicalTrials.gov (accessed on 30 December 2024) website). For example, a polysaccharide hydrogel (RadiaAce Gel) was used in a clinical trial for the prevention and treatment of breast cancer patients with grade 2 or higher radiation-induced skin injuries (NCT04481802). The skin is the largest organ in the human body, functioning as a barrier that protects against infection and harm. In the design of hydrogels for skin tissue engineering, flexibility and tensile strength are critical parameters for wound-healing applications, necessitating adjustments to accommodate bodily movements. The polysaccharide hydrogel exhibited a desirable adhesiveness and biocompatibility, flexible ductility, self-healing ability, and excellent antibacterial and angiogenic potential for the rapid healing of wounds [56]. Recently, a novel polysaccharide hydrogel (named the AFG/GelMA hydrogel) composed of glycosaminoglycan (AFG) and methylacrylylated gelatin (GelMA) was designed and synthesized using a covalent crosslinking method by the Wu M group [57]. Following a single application of the hydrogel to the skin wounds of diabetic rats and db/db diabetic mice, it dramatically enhances granulation tissue thickening, angiogenesis, and collagen deposition at the wound site. The Zhang M team developed a series of self-healable injectable conductive hydrogels for skeletal muscle regeneration in muscle tissue engineering [58]. The polysaccharide hydrogels were made using Schiff base reactions involving dextran-graft-aniline tetramer-graft-4-formylbenzoic acid and N-carboxyethyl chitosan. In vivo investigations on a muscle loss injury model demonstrated that these polysaccharide hydrogels significantly enhanced skeletal muscle tissue regeneration. Self-healable hydrogels are crucial for cardiac-tissue-engineering applications. Cell encapsulation with suitable biomaterials enhances cell viability and safeguards cardiomyocytes against injury [59]. Despite the implementation of many delivery techniques for treating cardiovascular disorders such as myocardial infarction, the majority of cells perish post-injection due to the adverse microenvironment characterized by inflammatory cytokines and detrimental free radicals [60]. Polysaccharide hydrogels have been shown to significantly enhance the adhesion, development, and proliferation of native heart cells following myocardial infarction. The cardiovascular regeneration application of the polysaccharide hydrogels should require extreme attention [61,62]. For example, Tarsitano M, et al. formulated chlorella-enriched alginate-gelatin (Alg-Gel) hydrogels for cardiovascular tissue engineering. In in vitro ischemia/reperfusion (I/R) models, chlorella-enriched Alg-Gel hydrogels reduced the reactive oxygen species production, as well as protected against myocardial damage [63]. Biomaterials and hydrogels are also crucial in the regeneration of brain tissue. Polysaccharide hydrogels have emerged as a viable delivery platform for neural cell growth, stem cell differentiation, and peripheral nerve conduit regeneration [64,65,66].

Based on a complex of polyacrylic acid and norbornene-functionalized chitosan, Hoang HT, et al. designed a dual pH-/thermo-responsive polysaccharide hydrogel using chemical crosslinking for colon-targeted drug delivery applications [67]. Through ionic crosslinking techniques, Fathi M and colleagues synthesized hydrogels with a dual responsiveness to temperature and pH by combining chitosan with poly(N-isopropylacrylamide-co-itaconic acid). These engineered materials, loaded with doxorubicin (DOX), demonstrated promising applications in localized breast cancer therapy while maintaining a low cytotoxicity [68]. For inflammatory bowel disease (IBD) applications, Laroui H and team engineered nanoparticles (NPs) designed to deliver the anti-inflammatory tripeptide Lys-Pro-Val (KPV) specifically to colonic tissue. Upon encapsulating the KPV-loaded nanoparticles within biocompatible polysaccharide hydrogels composed of alginate and chitosan, the polymers undergo degradation in the colon, thereby facilitating the targeted delivery of KPV-loaded nanoparticles to the site of inflammation [69]. You YC, et al. characterized a novel pH-sensitive hydrogel composed of konjac gum, xanthan gum, glycerol, and sodium alginate (KG-XG-GLY-SA hydrogel) that facilitates site-specific drug delivery to the colon. Their in vitro data demonstrated that synthetic polysaccharide hydrogels exhibited favorable colon-targeting characteristics. Moreover, in vivo data demonstrated that synthetic polysaccharide hydrogels can effectively treat ulcerative colitis without adverse side effects [70].

## 4. Polysaccharide Hydrogels in Tumor Immunotherapy

Inflammation is an adaptive process that occurred when the human body is exposed to the toxic stimuli. Owing to their favorable biodegradability, non-toxic, and biocompatibility properties, polysaccharide hydrogels have been widely applied as potential anti-inflammation and immunomodulatory biomaterials in medicine. The possible anti-inflammation mechanism of polysaccharide hydrogels mainly relies on regulating the inflammatory signaling pathway and pro-inflammatory cytokine production. Some polysaccharide hydrogels have been also shown to possess immunomodulatory properties, primarily through the activation of immune cells and the induction of immunity responses [71]. Because of the excellent biocompatibility and immunomodulatory properties in cancer immunotherapy, polysaccharide hydrogels were used as a good delivery platform in a variety of immunotherapy strategies for tumors.

Inflammation-related local metabolic disorders often lead to abnormal warming phenomena in tumor tissue. Polysaccharide hydrogels can reshape the metabolic program of immune cells and optimize the metabolic homeostasis of the tumor microenvironment (TME). In particular, they can interfere with important metabolic pathways such as aerobic glycolysis and the tricarboxylic acid cycle of macrophages, inhibit excessive metabolic stress, and, thus, reduce the abnormal heat production caused by inflammation. This creates a thermodynamic microenvironment that is unfavorable for the proliferation and survival of tumor cells and blocks the progression of tumors to a certain extent [72]. At the same time, the inherent immunomodulatory properties of polysaccharide hydrogels can directly act on the tumor microenvironment, activate tumor-infiltrating lymphocytes (TILs), and enhance their cytotoxic functions, effectively breaking the immune escape barrier of tumor cells, thereby inhibiting the growth and metastasis of tumors [73].

### 4.1. Polysaccharide Hydrogels in Adoptive T-Cell Therapy

Adoptive cell therapy has proven to be an effective approach in leukemia and lymphoma, but traditional delivery methods for therapeutic cells are always not satisfactory in treating solid tumors. Physiological barriers and immunosuppressive microenvironments of solid tumors are the major obstacle to adoptive T-cell therapy. The desirable biocompatibility and immunomodulatory properties have inspired researchers to attempt to apply hydrogel-based biomaterials in T-cell therapy. In experimental observation and practice, numerous studies have demonstrated that various hydrogels such as gelatin-based, self-assembling peptide, cationic polymer supramolecular hydrogels exhibit promising therapeutic effects in the delivery of adoptive T cells for the treatment of solid tumors [74,75,76,77]. As one kind of important biomacromoleules, polysaccharide has received extensive attention and is commonly used for hydrogel preparation. In tumor treatment, polysaccharide-based hydrogels were widely designed by researchers to deliver effective CAR-T immunotherapy.

**Figure 2:**
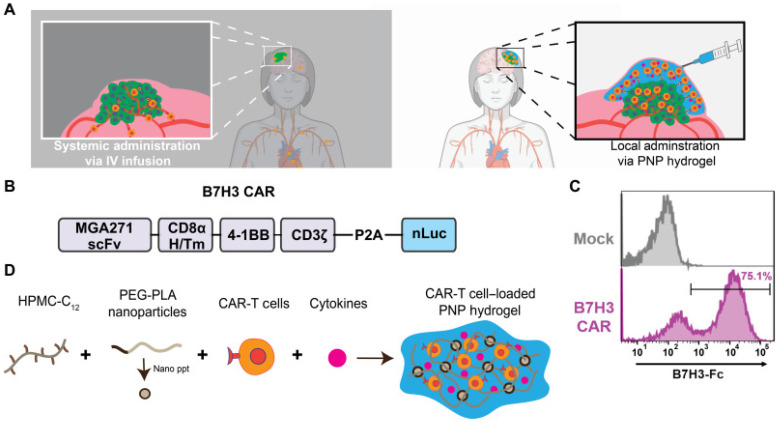
Schematic illustration of a proposed hydrogel for co-delivery of CAR-T cells and cytokines to treatment of solid tumors. (**A**) Schematic illustration showing the hydrogel delivery approach of CAR-T cells to solid tumors (right) in contrast to traditional intravenous (IV) methods (left). (**B**) Scheme of the B7H3 CAR construct employed in the research. (**C**) Validation of B7H3 CAR-T cells by flow cytometry compared to non-transduced mock T cells. (**D**) Design of CAR-T cell-loaded PNP hydrogels for the co-delivery of CAR-T cells and cytokines with HPMC-C_12_ and PEG-PLA nanoparticles. Reprinted with permission from Ref. [80]. Copyright 2022 Grosskopf AK, et al. Sci Adv.

Bhatta R, et al. presented a macroporous hydrogel composed of polysaccharides for in situ T-cell growth and improved anticancer effectiveness. Initially, DBCO-functionalized PEG and azido-functionalized alginate (Alg-N3) were produced in their study. To synthesize macroporous hydrogels, Alg-N3 was swiftly combined with DBCO-S-S-PEG, initiating a “click” reaction that resulted in the formation of a crosslinked hydrogel network. Unexpectedly, these T-cell-responsive macroporous hydrogels demonstrated anticancer activity comparable to traditional T-cell therapy with a reduced cell dosage [78]. Recently, various polysaccharide-hydrogel-based delivery techniques were developed to enhance the efficacy of CAR-T cells against solid tumors. Chao Y, et al. engineered a hydrogel scaffold for the co-delivery of metformin and CAR-T cells utilizing a natural polysaccharide, sodium alginate (ALG). Results from the tumor models indicate that metformin-infused hydrogels effectively enhance CAR-T therapy for both localized and distant abscopal tumors, while minimizing the systemic side effects [79]. The Appel EA laboratory has engineered an innovative hydrogel platform based on polysaccharides for the synchronized delivery of CAR-T cells and cytokine stimulants. The hydrogel formation occurs through self-assembly mechanisms, utilizing dodecyl-functionalized hydroxypropyl methylcellulose (HPMC) combined with biodegradable block-copolymer nanostructures. This delivery system optimizes CAR-T cell function by promoting their motility, persistence, viability, and activation, ultimately supporting the continuous production of cancer-targeting lymphocytes [80] (Figure 2).

The locoregional administration of immune cells using in situ forming hydrogel (ISFH) can increase the intratumoral localization and retention of CAR T cells by providing a cell scaffold suitable for controlled cell development, as has already been demonstrated with polysaccharide-based hydrogel implants [81,82]. In addition, ISFH could be used to protect cells during injection and then create an immunological niche by simultaneously encapsulating immunomodulating agents to improve cell viability [80]. As the reported results shown, the T-cell-loaded chitosan scaffold ISFH exhibited a favorable efficacy in cancer immunotherapy in the mice model [83].

### 4.2. Polysaccharide Hydrogels in Immune Checkpoint Inhibitors Treatment

Immune checkpoints are crucial regulators of the immune system, necessary for maintaining the functionality and responsiveness of T cells to diverse stimuli. Immune checkpoint drugs, primarily targeting CTLA-4, PD-1, and PD-L1, empower T cells to eliminate tumor cells [84]. These emerging immune checkpoint inhibitors (ICIs) have been proven as an efficient method to treat advanced cancers by inducing durable and systematic tumor-killing responses. However, in clinical treatment, many tumors could evade immune surveillance and exhibit a low response rate for ICIs. With the rapid development of Cas9/sgRNA technology and advanced biomaterials, researchers have developed a variety of methods to overcome the therapeutic shortcomings of immune checkpoint suppression [85]. Lee J, et al. developed a DNA aptamer hydrogel consisting of a PD-1 DNA aptamer and a sgRNA-targeting sequence that can be accurately cleaved by the Cas9 enzyme. In vivo, findings demonstrated that the co-injection of PD-1 DNA aptamer hydrogel (PAH) with Cas9/sgRNA produced markedly enhanced anticancer effects and survival rates. Furthermore, enhanced immune cell infiltration was noted in tumor tissues following PAH and Cas9/sgRNA therapy [86].

**Figure 3:**
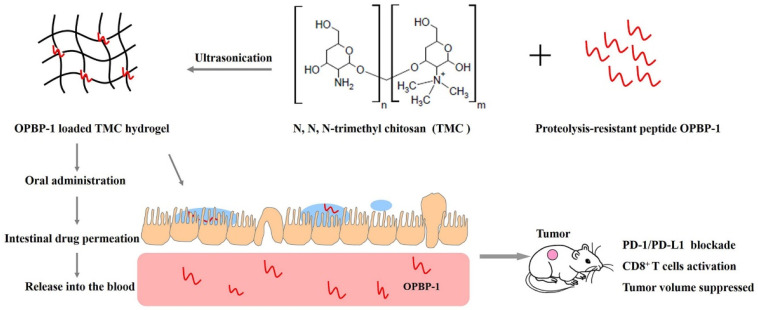
Schematic illustration of an orally available hydrogel for cancer immunotherapy. Reprinted with permission from Ref. [89]. Copyright 2021 Elsevier B.V. Li W, et al. J Control Release.

Immunotherapy utilizing a polysaccharide hydrogel to target checkpoint inhibitors, like CTLA-4 or PD-1/PD-L1, has emerged as an effective therapeutic strategy in cancer treatment. In a publication, Harui A, et al. encapsulated the anti-CTLA-4 antibody within a hydrogel matrix and administered it via peri-tumor injection to achieve the sustained perfusion of tumor-draining lymph nodes [87]. Others characterized an in situ crosslinked hydrogel for the prolonged release of antibodies that simultaneously target CTLA-4 and PD-1 signaling at the tumor site. This formulation yields an effective anti-tumor response with less systemic toxicity relative to the individual antibody delivery method, representing a strategy for immune checkpoint inhibitor cancer immunotherapy [88]. Li W and colleagues developed an oral delivery system for PD-L1 binding peptide (OPBP-1) using N,N,N-trimethyl chitosan (TMC) hydrogels. The resulting OPBP-1@TMC formulation demonstrated an enhanced oral bioavailability and significant tumor suppression in colorectal CT26 mouse models through PD-1/PD-L1 pathway inhibition [89] (Figure 3). While a dual-checkpoint blockade targeting PD-1 and CTLA-4 shows promise for advanced cancers, concerns persist regarding inflammatory and autoimmune complications [90].

Addressing these challenges, Harui A’s team engineered an injectable hydrogel system based on hyaluronic acid (HA) for anti-CTLA-4 antibody delivery. Their synthesis protocol involved the separate preparation of thiolated carboxymethyl hyaluronic acid (CMHA-S) and poly(ethylene glycol) diacrylate (PEGDA) solutions, followed by the sequential incorporation of anti-CTLA-4 antibodies into PEGDA and a subsequent CMHA-S addition to initiate gelation. This localized delivery approach, when combined with systemic anti-PD-1 treatment, effectively controlled tumor growth and generated durable responses evidenced by the protection against a distant tumor rechallenge [91].

### 4.3. Polysaccharide Hydrogels in Cancer Vaccine Therapy

Cancer vaccines are crucial in eliciting T-cell-mediated tumor cytotoxicity. In this mechanism, cancer vaccines initially provide adequate antigens to the antigen-presenting cells (APCs) to stimulate anti-tumor immunity. Furthermore, immunoadjuvants and chemokines may be administered to attract APCs to enhance antigen presentation. Thirdly, APCs, such as macrophages (MФ) and dendritic cells (DCs), process the collected antigens and travel to the lymph nodes. Ultimately, the T cells differentiate into effector and memory T cells, with the effector T cells migrating to the tumor site to eliminate the tumor cells [92]. Cancer vaccines can be classified according to the kind of administered antigens, including DNA/RNA vaccines, DC vaccines, and peptide vaccinations [93]. The efficacy of vaccinations is significantly reliant on the continuous exposure to antigens and the long-lasting cellular immune responses [94]. Various drug-delivery methods, including cationic polymers, liposomes, polysaccharides, and nanoparticles, can transport therapeutic agents and elicit a protective immune response [95]. The loaded antigens experienced continuous degradation and a loss of immunogenicity with alterations in the pH, temperature, or reaction conditions. Consequently, there is an immediate want for delivery methods capable of releasing unaltered antigens. Hydrogels serve as an exceptional choice for delivery methods due to their superior biocompatibility and substantial loading capacity [96].

Successful therapeutic vaccination requires tumor antigens that have sufficient immunogenicity and a strategy to overcome the mechanisms of tumor immunosuppression [97,98]. Hydrogels allow the prolonged concurrent administration of antigens, including peptides, nucleic acids, cell lysates, and adjuvants, thus mimicking the priming and strengthening of traditional multiple-injection protocols [99]. They can also be given together with other immunomodulating agents to combat immunosuppression (the ideal state has yet to be determined).

**Figure 4:**
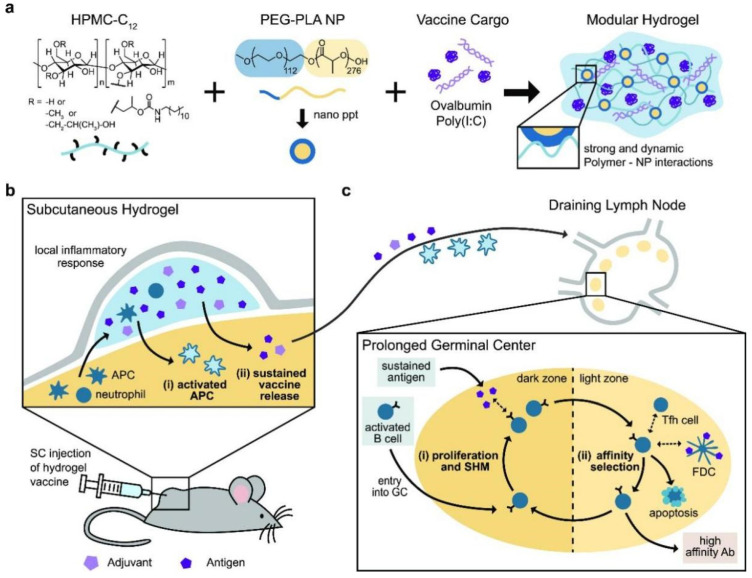
Schematic representation of the PNP hydrogel in vaccine delivery. (**a**) Vaccine-loaded hydrogels were synthesized by the combination of HPMC-C_12_, PEG-PLA nanoparticles and vaccine cargo. (**b**) Vaccine-loaded hydrogels were subcutaneously injected into mice for local inflammatory response, resulting in activated antigen presenting cells (APCs) and sustained vaccine release. (**c**) Changes of the prolonged germinal center, where increased antigen availability in turn promote the development of more selective antibodies and enhanced immune response. Reprinted with permission from Ref. [105]. Copyright Roth GA, et al. ACS Cent Sci.

Researchers developed a series of polysaccharide hydrogels for vaccine administration in immunotherapy. Fan B et al. described the creation of a new dextran-based hydrogel intended as an injectable depot for peptide vaccines. The hydrogel has self-healing properties as a result of the dynamic covalent crosslinks produced by the double Michael addition of thiols to alkynones. These injectable, non-toxic hydrogels demonstrate a significant loading capacity and a regulated release of the unmodified antigen, resulting in T-cell activation and a prolonged immunological response [100]. Polysaccharide hydrogels laden with immunomodulators serve as a prevalent technique for recruiting dendritic cells as vaccine carriers. Glycoconjugates ending in mannose, fucose, or N-acetylglucosamine serve as natural ligands extensively utilized in dendritic-cell-targeting malignancy therapy [101]. Bencherif SA and colleagues developed a hydrogel system made from alginate for vaccination therapy in melanoma. The hydrogel was made using methacrylated alginate infused with cytosine-phosphodiester-guanine oligodeoxynucleotide (CpG ODN, an immunological adjuvant) and GM-CSF (a cytokine adjuvant), resulting in a substantial capacity for vaccine delivery to elicit a robust and long-lasting T effector response [102]. Likewise, Yang F, et al. developed a biodegradable thermosensitive hydrogel vaccine that encapsulates CpG ODN and GM-CSF to facilitate the continuous release of immunomodulators and antigens [103]. CD40 agonist antibodies may augment the tumoricidal activity of T lymphocytes by targeting dendritic cells. A controlled-release hydrogel delivery method containing an immunostimulatory CD40 agonist significantly enhanced treatment efficacy in the B16F10 melanoma model [19]. Additional researchers employed the self-healing polymer-nanoparticle (PNP) hydrogel platform to target DCs in tumor immunotherapy [104,105] (Figure 4). Consequently, the development of designing polysaccharide hydrogels capable of attracting immune cells through various pathways is anticipated to be a trend in tumor vaccine therapy.

### 4.4. Polysaccharide Hydrogels in Oncolytic Virus Treatment

The concept of virotherapy for malignancies has brought about a huge evolution in tumor therapy. Oncolytic viruses (OVs) primarily function by eliciting antiviral immune responses that target and eradicate infected tumor cells. The efficacy of oncolytic viruses in tumor therapy mostly relies on their capacity to reach tumor cells without detection by the host’s immune system. Diverse approaches and processes, including the utilization of nanoparticles (NPs), have been implemented for the delivery of OVs to tumor cells [106].

**Figure 5:**
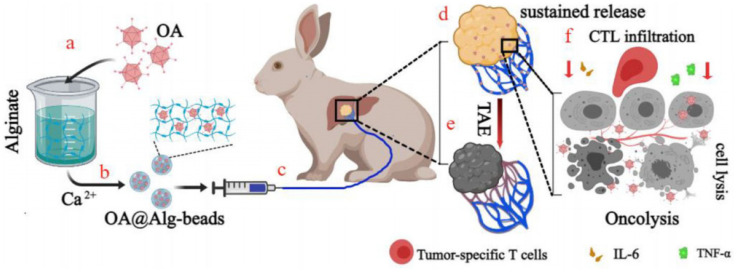
Schematic illustration of the TAVE strategy. (**a**,**b**) The formation of OA@Alg-beads. (**c**) OA@Alg-beads are administered through the liver arteries. (**d**) In tumor region, OA sustained release from OA@Alg-beads. (**e**) Tumor cells apoptosis. (**f**) OA@Alg-beads induce the intensive immune response and infiltration of T cells. Reprinted with permission from Ref. [110]. Copyright Peng Lv, et al. Advanced Therapeutics.

Hydrogels are particularly appropriate for oncolytic virus delivery therapy because of their biodegradable, biocompatible, and nonimmunogenic characteristics. Le TMD and colleagues developed a set of dual pH-and-temperature-responsive physically crosslinked injectable hydrogel systems for the effective and sustained delivery of oncolytic adenovirus (OA). These hydrogels demonstrated significant cytotoxicity against cultured cancer cells and had an improved anti-tumor therapeutic efficacy in xenograft tumor models [107]. A study by the Yun CO group enclosed a TNF-related apoptosis-inducing ligand (TRAIL)-expressing oncolytic adenovirus (oAd-TRAIL) in hydrogel (oAd-TRAIL/gel) to augment and extend the anticancer activity of the virus. The results indicate that the oAd-TRAIL/gel effectively induces apoptosis and inhibits cell growth in malignancies [108]. To maximize the potential of OA-mediated virotherapy, Choi J W, et al. developed a sustained injectable alginate hydrogel delivery system. As described in the results, OA encapsulated in an alginate-based hydrogel showed greater anti-tumor activity than a single OA injection in human xenograft tumor models [109]. In the hepatocellular carcinoma treatment, the Liu G group described a novel calcium alginate hydrogel microsphere with OA encapsulated (OA@Alg beads) in transcatheter arterial viroembolization (TAVE) strategy to enhance and prolong the anti-tumor efficacy of oncolytic adenovirus [110] (Figure 5). Moreover, polysaccharide-based hydrogels may have great therapeutic potential in the combination therapy of the oncolytic virus with immune checkpoint inhibitors and other immunotherapies [111].

### 4.5. Polysaccharide Hydrogels in Combined Immunotherapy

The tumor microenvironment is the key obstacle for the anti-tumor immune response that must be recognized by researchers when developing novel immunotherapies. Hence, immunotherapeutic approaches with the aim of making these “cold” tumors switch into “hot” tumors are urgently needed. Combinational immunotherapeutic strategies represent a rapidly developing frontier in tumor immunotherapy [112]. For one thing, a combination of different forms of immunotherapy can maximize the immune cell response within the tumor tissue. A combination immunotherapy utilizing CAR T cells and checkpoint inhibition has demonstrated significant efficacy in the treatment of solid cancers. In the mechanism, CAR T cells facilitate infiltration, but the PD-1/PD-L1 blockage guarantees a prolonged persistence and functionality [113]. Moreover, interference with the PD-1/PD-1 pathway with PD-1 checkpoint inhibitors or shRNA inhibition may restore the effector function of CAR T cells [114]. Another combinational strategy is the combination of tumor immunotherapy with chemotherapy or radiotherapy, which compensates for each other and more efficiently inhibits the cell proliferation and cell growth in tumors [115]. To date, the combination of immunotherapy with chemotherapy has become the broadest treatment option for a variety of tumors in clinic. In a double-blind phase III trial, the combination of the PD-1 inhibitor pembrolizumab with pemetrexed and platinum chemotherapy as the first-line treatment for NSCLC without EGFR or ALK mutations yielded significantly prolonged overall survival and progression-free survival in patients [116].

**Figure 6:**
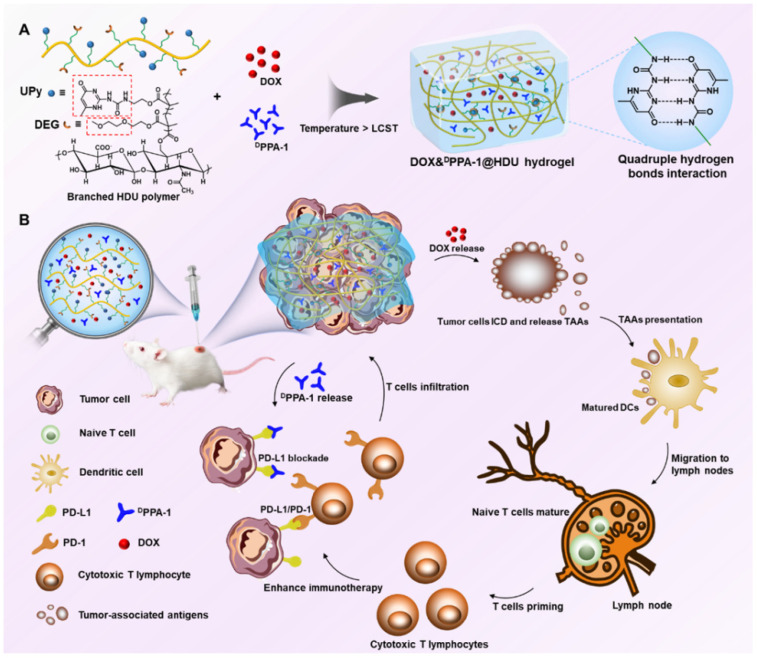
Schematic illustration of an injectable hyaluronic-acid-based supramolecular hydrogel for co-delivery of DPPA-1 and DOX. (**A**) Design of DOX&DPPA-1@HDU hydrogel with DPPA-1 and DOX. (**B**) The internal immune responses of DOX&DPPA-1@HDU hydrogel in mice. Reprinted with permission from Ref. [118]. Copyright Liu M, et al. ACS Appl Mater Interfaces.

Due to their unique advantages as mentioned above, polysaccharide hydrogels have been increasingly used in applications of multiple immunotherapy-based combinational strategies. A work by the Gu Z group produced a biodegradable hyaluronic-acid-based hydrogel to encapsulate CAR-T cells targeting chondroitin sulfate proteoglycan 4 (CSPG4) for implantation in a melanoma-tumor-bearing mice model. The hydrogel was infused with the cytokine IL-15 to preserve the activity and proliferation capacity of CAR-T cells, while the anti-PDL1-blocking antibody (aPDL1) was included to inhibit the PD1/PDL1 pathway. The engineered hydrogel establishes a favorable environment for CAR-T cells to eliminate tumor cells during surgery and inhibit tumor recurrence [82]. To enhance the therapeutic efficacy of vaccines and immune checkpoint inhibitors, Song H, et al. developed an injectable PEG-b-poly(L-alanine) hydrogel for the co-delivery of a tumor vaccine including tumor antigens/GM-CSF and dual-immune-checkpoint inhibitors (anti-CTLA-4/PD-1 antibody). The results indicate that this hydrogel-based combinatorial immunotherapy markedly enhanced tumor-infiltrating CD8+ T cells and substantially improved the efficacy of immunotherapy in malignancies [117]. Liu M et al. presented an injectable hyaluronic-acid-based supramolecular hydrogel to improve chemo-immunotherapy in cancer by delivering the DPPA-1 peptide, a D-peptide antagonist with a high binding affinity to PD-L1, and doxorubicin (DOX). DOX can directly eliminate malignant cells and trigger apoptosis. The DPPA-1 peptide may inhibit the PD-1/PD-L1 pathway, hence enhancing the T-cell-mediated immune responses and reducing adverse effects [118] (Figure 6). Polysaccharide hydrogels may also be utilized in the combinatorial approach of radiation and immunotherapy. Shen W, et al. developed a sodium alginate hydrogel incorporating elesclomol-Cu and galactose to facilitate cuproptosis (Cu^2+^-mediated cell death), thereby augmenting radio-immunotherapy in colon cancer. The polymeric hydrogel effectively induced cuproptosis, considerably enhanced the sensitization of radio-immunotherapy, and extended the survival of tumor-bearing mice in both local and metastatic colorectal cancer models [119].

## 5. Challenges and Future Perspectives

Hydrogels are a category of polymeric materials characterized by a three-dimensional network structure, capable of absorbing substantial quantities of water and biological fluids. Polysaccharides, being natural polymers with superior biochemical and physical properties, have garnered significant interest for their essential medicinal uses, particularly as scaffold materials in cancer therapy [120]. This review summarizes and analyzes polysaccharide hydrogels with diverse architectures and unique bioactivities, highlighting their exceptional efficacy in tumor immunotherapy and elucidating their immunomodulatory mechanisms.

An optimal tumor-targeted drug delivery system would differentiate between tumor and healthy tissues while accurately, and in an on-demand manner, regulating therapeutic drug dosages. Hydrogels exhibit biocompatibility and biodegradability in vivo. The loose and porous structure is optimal for pharmaceutical loading, whereas swelling and sophisticated environmental response mechanisms can control the quantity and rate of drug release. Incorporating natural polysaccharides into hydrogel systems may facilitate the safe and effective treatment of tumor-specific diseases.

Although polysaccharide hydrogels exhibit significant immunotherapeutic promise, it is essential that we acknowledge that much progress is still required to translate these discoveries into clinical practice. Some concerns may need to be taken into consideration before clinical usage. These include the development of efficient manufacturing processes, the optimization of hydrogel properties, and the identification of suitable therapeutic payloads. In manufacturing processes, the extraction and purification of functional polysaccharide should be taken into consideration. Natural organisms serve as the primary source of polysaccharides; nevertheless, effective extraction procedures to obtain high-purity polysaccharides remain inadequate [121]. Furthermore, the numerous modification sites on polysaccharide molecules complicate the acquisition of polysaccharides with specific chemical structures. The optimization of hydrogel properties is another bigger question. For instance, most of the polysaccharide hydrogel is hydrophilic, which is suitable for a carrier of hydrophilic drugs. However, this polysaccharide-based hydrophilic hydrogel delivery system showed poor performance when used to load hydrophobic drugs. How to increase the hydrophobicity of the hydrogel to deliver hydrophobic drugs into tumors is still a big deal. Nanocomposite hydrogels have recently emerged by combining the benefits of hydrogels and enhancing their physicochemical properties, demonstrating a controlled release capability, favorable EPR effect, extended immune response duration, and increased efficacy in tumor therapy [122].

Considering that the polysaccharide hydrogels demonstrated excellent properties in terms of natural sources, biodegradability, and non-toxicity, and exhibited good biocompatibility, they are suitable candidates for potential use in tumor immunotherapy applications [123,124]. Future research should focus on the in vivo and clinical bioactivity studies of drug-loaded polysaccharide hydrogel, with an in-depth investigation of their pharmacokinetics under various cancer microenvironment.

In conclusion, polysaccharide hydrogels have surfaced as a viable platform for immunotherapy, with distinct features like biocompatibility, malleability, and adjustable degradation. Recent improvements in the manufacture and modification of these hydrogels provide significant potential for the creation of innovative immunotherapies with an improved efficacy and diminished side effects. Additional investigation into polysaccharide hydrogels is anticipated to offer a novel perspective for the development of innovative tumor immunotherapy techniques that may lead to significant advancements in clinical practice.

## Figures and Tables

**Table 2:** Polysaccharide hydrogels and their application in clinical trials.

Registration Number	Clinical Applications
NCT04481802	Breast cancer patients with skin injuries
NCT05082480	Adhesion occurrence after trigger finger release surgery
NCT06447311	Papilla augmentation
NCT05853224	Treatment of soft tissue deficits
NCT06492811	Treatment of diabetic wounds
NCT06039774	Recurrent aphthous stomatitis
NCT06758440	Modulation of osseointegration
NCT06373757	Non-surgical treatment of intrabony defect
NCT03754010	Periodontal treatment
NCT04377256	Xenogenic bone healing
NCT04740086	Treatment of cryptoglandular fistula-in-ano
NCT03321396	Assist endoscopic submucosal dissection
NCT06584617	Expedite chronic diabetic foot ulcer healing

Note: Data from ClinicalTrials.gov (accessed on 30 December 2024) website.

## Data Availability

No new data were created or analyzed in this study.
